# Police officers’ perceptions of a training course designed to enhance open-ended questions with adult witnesses

**DOI:** 10.1080/13218719.2024.2346718

**Published:** 2024-09-01

**Authors:** Hamida Zekiroski, Martine B. Powell, Kate Cashman

**Affiliations:** a Centre for Investigative Interviewing, Griffith Criminology Institute, Griffith University, Mount Gravatt, QLD, Australia; b School of Social Sciences, University of Tasmania, Hobart, TAS, Australia

**Keywords:** adult interviewing, best-practice interviewing, investigative interviewing, police, questioning, training evaluation

## Abstract

This study evaluated police officers’ perceptions of a new training program aimed at improving interviewing skills for adult interviewees. Drawing from successful strategies used in interviewing child complainants of abuse, the program focused on enhancing the use of open-ended questions. The investigation aimed to identify effective aspects, limitations, the practicality of open-ended questions in real-world scenarios, and avenues for enhancing post-training interviewer performance. Through diverse qualitative data collection methods including email feedback, discussion boards, questionnaires, and follow-up interviews, officers’ feedback was thematically analysed. Four key themes emerged: the utility of open-ended questions, improved skills due to course content, variability in narrative detail needs across contexts, and opportunities for enhancing learning outcomes. These findings have implications for both research and practical applications, highlighting the effectiveness of the training program and suggesting areas for further improvement in enhancing police officers’ interviewing skills with adults.

The current study is a qualitative evaluation of a new training programme designed to enhance open-ended question usage among generalist police who elicit verbal evidence from adult witnesses. Interviews with witnesses are conducted in all areas of policing, and it is well established that open-ended questions are a core skill in conducting these interviews effectively (Powell et al., 2019). The essence of effective interviewing lies in establishing a trusting relationship, ensuring witnesses feel fairly treated, without judgment, and granting them the flexibility to respond in a way that accurately reflects their recollection of events (Fisher & Geiselman, 1992). Unlike specific cued-recall questions (e.g. ‘who’, ‘what’, ‘when’, ‘where’) and closed questions (e.g. ‘do you know who this person is?’), which tend to elicit short-answer responses focused on precise detail, open-ended questions (e.g. ‘Tell me what happened’, ‘What happened next’) cast the net wide and allow respondents to report what they remember in narrative format (Powell & Snow, 2007; Snook et al., 2012). When used well, open-ended questions improve the accuracy and detail of verbal evidence, elicit contextual detail, enhance witness credibility, reduce defensiveness and anxiety, and counteract witnesses’ tendency to withhold information (Brubacher et al., 2019; Pichler et al., 2019; Powell & Cauchi, 2013). Consensus around the importance of open-ended questions is illustrated by the consistency and similarity of various interview protocols that are all focused on prioritising narrative detail – for example, the National Institute of Child Health and Human Development (NICHD) protocol (Lamb et al., 2018), the Standard Interview Method (SIM; Powell & Brubacher, 2020), the Guidance for Achieving Best Evidence (ABE) in Criminal Proceedings (Ministry of Justice, 2011), and the Cognitive Interview (Fisher & Geiselman, 1992).

Despite scientific agreement that open-ended questions are a critical component of good investigative interviews, research around the best way to teach these questions in a sustainable way is still in its infancy. Good interviewing (like any complex skill) evolves from scientific enquiry, the systematic study of interviewer and witness behaviour, the evaluation of new improved methods against agreed outcomes, and the establishment of mechanisms to transmit new knowledge and promote change in practice. So far, it has been established that mastering the skill of open-ended questioning requires an incremental approach (Brubacher et al., 2022). This process encompasses various tasks, including the capacity to recognise various types of questions and their impact (Powell et al., 2016; Yii et al., 2014), learning different question stems proficiently (Powell & Wright, 2008), engaging in spaced practice while utilising open-ended questions within simulated interview scenarios, where interviewers are exposed to common challenges (Hughes-Scholes & Powell, 2013), and receiving expert feedback tailored towards rectifying behaviour and generating alternative strategies (Powell et al., 2022). While researchers are still refining the precise elements and how they should be delivered, their importance has been established across many contexts including research involving generalist police who interview adult witnesses. Specifically, Zekiroski et al. (2024) evaluated a training programme developed for generalist police that included all the key training activities listed above. Interviewer performance (as measured in simulated interviews where a researcher played the role of an interviewee) increased over time from baselines of 6–18% open-ended questions to 70–76% following the programme. Further, the study revealed little drop in performance at least 6-months after the training ceased with no intervening follow-up training sessions.

Quantitative research comparing rates of open-ended questions pre- versus post- training is important in establishing the efficacy of training; however, it needs to be accompanied by other research methods, including analysis of qualitative feedback from police officers who attempt to implement learned strategies in the field. The perspectives of police who administer questioning techniques on a practical level is helpful for identifying factors that constrain or enable transfer of learning into the workplace and how the interviewer training programmes could be improved. Indeed, it is not uncommon for implementation gaps to occur at the point of service delivery, often with consequences that are unforeseen by the policy developers (e.g. Hill & Hupe, 2002; Lipsky, 2010). Open-ended question usage tends to be lower in the field than in the ‘simulated’ training setting, which suggests that there are potential challenges embedding these questions within certain types of case work (Benson & Powell, 2015; Navarro et al., 2024; Powell, 2002).

Police officers’ elaborate feedback regarding the investigative interviewer training courses could be helpful in several ways. First, the feedback may provide insight onto how organisational factors such as workload and supervisor expectations impact trainees’ ability to apply the interview strategies (Benson & Powell, 2015; Wright et al., 2006). Second, officers’ reflections on the value of the training activities provide insight into the degree to which the activities sparked intrinsic motivation, and whether they enjoyed exploring the material and felt they mastered it (Hauer et al., 2011). Third, officers’ feedback could provide insight into any misunderstandings that arose from the course, in terms of both the content taught and expectations regarding the application process. Each of these elements is important for quality improvement.

In conclusion, this study elicits the anonymous perspectives of a group of generalist police officers who successfully completed a training programme that focused on the use of open-ended questions when eliciting evidential detail from adult witnesses. Specifically, the officers had undergone the same programme that, in previous research, was shown to lead to 70–76% open-ended question usage in simulated interviews (Zekiroski et al., 2024). The feedback, perceptions and experiences of those police who completed the course were collated using a variety of qualitative data collection methods (e.g. observations via email to the course coordinator, anonymous discussion boards and an evaluation questionnaire). The officers’ perceptions were used to provide insight into why the programme worked, its limitations, the utility of open-ended questions in the field and how interviewer performance post-training could potentially be further improved.

## Method

### Participants

The study was approved by the Human Research Ethics Committee at Griffith University, as well as the executives of the police organisation. The participants included 174 police officers who enrolled in the investigative interview training course between February 2018 and November 2019. Ten people did not complete the course for various reasons (e.g. personal circumstances, professional transfers within the department, resigned from the academy). Police officers included recruits (who had just joined the police and were doing the course while at the academy, *n* = 60), frontline police (*n* = 55) as well as officers who were detectives or studying to be detectives, hereby referred to as detectives (*n* = 40). The final sample included 155 officers who spanned equally across the jurisdiction. The officers (frontline and detectives) varied in ranks from Constable to Senior Sergeant.

### Materials and procedure

#### Course structure and content

The course that the officers completed in the current study was evaluated by Zekiroski et al. (2024). It took approximately 38 hours to complete (spread over 6–12 weeks) and consisted of 11 modules, which focused on developing officers’ understanding of the effect of various questions and being able to use these effectively. Considerable attention was focused on the most challenging skill – understanding subtypes of open-ended questions and being able to use them effectively to elicit a narrative account of an offence. Initial invitations encourage interviewees to recall details about an event, often prompting them with phrases like ‘Start from the beginning . . . ’ or ‘Tell me everything you can remember about what happened . . . ’. Research indicates that these invitations are effective in eliciting abundant and accurate information (Feltis et al., 2010; Kontogianni et al., 2020; Orbach et al., 2000). Breadth prompts, such as ‘Then what happened?’ or ‘What else happened?’, prompt interviewees to recount additional events or sub-events related to the main incident. Depth prompts, on the other hand, probe deeper into specific details mentioned by the interviewee, such as ‘You said X, tell me more about that’ or ‘What happened when X’, where X represents a pre-disclosed detail.

The officers progressed through set activities in the course at their own pace, alongside six simulated practice sessions with a tutor. The Appendix (Zekiroski et al., 2024) provides an overview of the training programme content and structure, which was identical for all police. All officers were expected to complete the course during work hours; for the recruits (who were based at the academy) that time was set by the academy as opposed to the officer.

The practice sessions were conducted over the telephone, with trained research assistants and fellows who played the role of the interviewees (witnesses or complainants) using a range of pre-prepared scenarios, such as a family violence incident, robbery, traffic accident, physical assault and fraud. These provided an opportunity for the officers to demonstrate skill in eliciting a detailed and accurate account for various offences for evidential purposes and to elicit feedback. Before the mock interview session, the officers received an email containing a brief description of the scenario. For example:

*You are called to a private address in [Street Name] Street, [Suburb] by the radio room as the owner has reported a break in. Upon arrival you see that a panel of glass in the front door has been smashed. The resident, Ash, answers the door when you knock.*

All role-players were trained to ensure consistency in their responses. For instance, when faced with an open-ended question, the role-players were instructed to provide three or four relevant details in their response. If a question was designed to elicit specific information (e.g. ‘who’, ‘when’ ‘where’, ‘why’ questions), the role-players provided one piece of information, and yes/no questions were always responded with ‘yes’ or ‘no’, including ‘Can you tell me . . . ?’ or ‘Do you remember . . . ?’ questions. This training approach, as outlined by Powell et al. (2022), was implemented to ensure fairness across learners, reward ideal question types (i.e. shape performance) and control the amount of information provided across scenarios. Regardless of the scenario, the quantity and quality of information were standardised for all trainees, and the purpose was to practise eliciting an accurate and detailed account of the allegation.

#### Method of eliciting feedback

The feedback, perceptions and experiences of those police who completed the course were collated using a variety of qualitative data collection methods. Firstly, trainees were invited at any time to provide written feedback expressing concerns, queries or observations via email to the course coordinator who was not a police staff member. Secondly, discussion boards at the end of each module of the course invited the officers to provide comments on their ‘thoughts so far’. This discussion board was a group forum to share thoughts on the course content, ask questions and interact with fellow students. Thirdly, course feedback was delivered via a standardised questionnaire at the end of the course and the follow-up interview with trainers who were not police personnel. Any feedback related to the course and its utility was also included in the text analysis.

#### Data management and analysis

Feedback focused on the utility of the course (particularly training in use of open-ended questions) as well as the course structure and how the activities and the trainee experience might be improved. All data were collected and de-identified prior to analysis. A total of approximately 107,900 words of transcript were compiled. Braun and Clarke’s (2006, 2012) approach to thematic analysis was deemed the most suitable approach for the data analysis. The first author adopted an iterative process as recommended by Braun and Clarke (2012). This involved data immersion through the reading and re-reading of transcripts and listening to the audio recordings if they were available. This allowed familiarisation with the content and allowed notation of initial analytic observations (Phase 1). The data were then systematically coded, and initial codes (units of meaning) were generated (Phase 2). Broader patterns of meaning, referred to as 'themes', were developed inductively with the second author based on word groups (Phase 3). These themes were subsequently reviewed and refined to ensure the relevance of text allocated to each theme (Phase 4). Theme names were generated to identify the 'essence’ of each theme and to confirm that the data had been adequately captured (Phase 5). Finally, the themes were integrated into an analytic narrative of the police’s feedback, and the findings were reported in the current paper (Phase 6). The quotes provided to illustrate the results of this study have been edited for grammatical correction and readability and to de-identify all participants.

## Results

Four multifaceted themes were generated to represent the officers’ feedback. These themes were labelled as follows: open-ended questions have immense utility in the field; the improved skills are attributed to course content and structure; the need for narrative detail is not consistent across interview contexts; and learning outcomes could be further improved. Each of these themes is now summarised in turn.

### Open-ended questions have immense utility in the field

All officers (irrespective of their rank, career stage and field of policing) indicated that, as a result of the course, they felt confident using open-ended questions, they understood the value of these questions and were now regularly using the skills in the field. Responses about the value of the course were highly enthusiastic, as illustrated in the quote below.

In my time as a Detective of an Investigation Branch, I was never exposed to such a level of learning. I am not in an investigative role right now, but I am in a Supervisory role which allows me to educate, mentor and guide junior constables. This course has provided me with the necessary tools for my job, a great understanding of the importance of open-ended questioning to elicit a free, elaborate and detailed response, and an understanding of the necessity of these questions in a range of investigations, regardless of seriousness. (Detective)

Coming from a law background I generally used only specific questioning skills to ascertain direct information relevant to a case and to reduce time allocated to specific tasks. Throughout this course, my questioning skills and style have changed dramatically. The techniques learned, especially exhausting a narrative, is critical in policing and I believe this course has sufficiently developed these skills. (Recruit)

Given their better understanding of open-ended questions, the officers perceived they were now better equipped to gather critical evidence to support charges, and that the increase in narrative detail about what happened would likely be helpful for prosecution. Further, the benefit of skills in eliciting narrative detail was not constrained to witnesses, or to audiovisual testimony. The analysis of feedback reflected broad support for the utility of open-ended questions; the question type was deemed useful across all types of interviews, including those with offenders and interviews conducted for the purpose of eliciting a written statement.

I used my skills to interview an offender. I just let him say whatever the hell he wanted to say, in narrative detail. In telling his story, he was getting really wound up, and his manner was aggressive. When the case went to trial, his interview and manner actually worked against him and favoured the prosecution. That wouldn’t have been the case if he’d given a series of short answers to closed questions. (Frontline officer)

Knowing how to elicit a free narrative makes a massive difference when you are interviewing crooks who are willing to tell their story. It gives you the material to match against hard evidence; to say, for example ‘You just told me X, but that doesn’t match Y’. It’s actually really fun when you can do this! (Detective)

From doing this course, I’ve changed the way I take written statements. Before I used to ask a series of specific questions up front, but I can see that by doing that, I was just putting what I *thought* they were saying into their statements. Now I type exactly what they’re saying, and then come back and ask those specific questions at the end. I get all of the information first with open-ended questions so as not to influence their story. (Detective)

### Improved skill is attributed to better understanding of open-ended questions

Although the frontline officers and detectives had received training previously using a protocol that emphasised narrative detail, they did not perceive they had acquired the skills to sustain open-ended question usage in the field prior to this course. Some of the feedback from officers indicated that the reason for not using open-ended questions previously was that they defined them differently. Specifically, they had defined ‘Wh’ questions (i.e. specific cued-recall questions) as open-ended, when in reality ‘Wh’ questions do not elicit narrative detail.

I really didn’t want to do further investigative interviewing study – I was forced to, in order to get promoted to detective level. When I started, I didn’t think I’d learn much. But hand on my heart, I now wish I had done it 10 years ago. Previously, I was only taught to use specific questions; ‘where’, ‘how’, ‘why’, etc. I thought that was the bee’s knees. When I did this course, I realised I’d been doing it wrong for so many years. (Detective)

I have been a police officer for over 38 years and was only ever taught to use specific questions. I have now changed my entire view on interviewing. With the knowledge I have gained from doing this course, I now use open-ended questions to gain as much information as possible first. That’s how it should be – to hear what the person has to tell me, not the bits that I think are important. I want to hear *their* answer; it is about them, not me! (Detective)

Prior to this course, I thought I had a reasonable understanding of the free narrative style of interviewing. Turns out, I was wrong! Previously I did try to encourage narratives at the beginning of an interview but wasn’t sure how to obtain further information without resorting to specific questioning. I now see the detrimental effect of these questions, especially on interviewee credibility. I think I was overly concerned with gathering every detail regardless of whether it was relevant to the investigation, or its effect on the interviewee. (Detective)

Of all the tasks, the officers attributed two types of course activities to be most beneficial. This included the coding task, where they needed to learn and identify various questions within transcripts and conducting simulated mock interviews with the assistant researcher playing the role of respondent. These activities were deemed beneficial, albeit not always enjoyable.

While I did find the coding activities to be long and arduous, I now have a better understanding of the different types of questions and how they are categorised and defined. (Recruit)

The least enjoyable activity for me was the coding exercise but seeing how the different questioning styles impacted on the interviewee’s answers helped. (Detective)

I think I learnt the most from the practical mock interviews. Although I didn’t enjoy doing them as I was worried about doing poorly, at the end of each interview I felt more confident that I had understood the theory components of the modules. I found it helpful that the assessors provided feedback immediately after all the mock interviews. This helped me to modify my style of questioning whilst the interview was still fresh in my mind. (Detective)

### The need for narrative detail is not consistent across interview contexts

The value of using open-ended questions to obtain narrative offence-related detail was not perceived to be equal in all policing contexts. Specifically, the utility of the open-ended questions was deemed most important in cases where a complainant is the only or main witness to what occurred (this is common in family violence and sexual offences). Open-ended questions were deemed least important in situations where there is sufficient physical evidence to support a charge (e.g. CCTV footage capturing the offender in action). For some crimes, a complainant or witness would have little narrative detail to report because they were not present when the offence occurred (e.g. a person’s belongings may have been stolen when they were not on their premises). Safety issues also needed to be considered; for some traffic accidents it was perceived to be inappropriate to prioritise getting a narrative over ensuring the safety of the people involved. Finally, officers’ feedback indicated that the type and range of evidence also needed to be considered. For example, with drug trafficking offences, story detail may be less relevant once it is established who supplied the drugs and the intent behind acquiring them.

It would be too time consuming and unsafe to use open-ended questions for the crash report scenario we just did. You’ve got vehicles blocking traffic so technically they are in the way and it’s not operationally safe to hang out there taking a report. You can also see the scene yourself as a police officer, so you have a fair idea of what’s going on. Shoplifting is another example where you don’t need to go into a detailed narrative with open questions because you can see the CCTV footage and the shop owner knows what’s missing. So, I would say an extensive narrative is less important for property and incident-based crimes but more important for crimes like family violence, assaults, breach of orders, particularly when you have a bit more time to deal with it. (Frontline officer)

Nonetheless, the officers’ feedback revealed that the utility of open-ended questions should be assessed on an individual case-by-case basis as opposed to crime type. Even for seemingly simple cases, a narrative account may reveal that the case is a lot more complex than initially perceived. Further, while an offender may initially appear non-cooperative (i.e. saying ‘no comment’), carefully crafted open-ended questions focused on getting their ‘side’ of the story can build rapport and subsequently lead to extensive detail. Officers emphasised the importance of being open-minded about what occurred and about the skill level of the interviewee, so that they were prepared to use a combination of questions in *all* cases where open-ended questions were needed.

Recently I started using open-ended breadth and depth questions with a vulnerable suspect who I was interviewing for a simple breach of Restraining Order by phone. She went on to provide further details that she had planned to stab the victim and immediately prior to police involvement had placed the knife in her handbag and was drinking alcohol to gain the courage to implement her plan. I am confident that if I had interviewed her prior to completing this course I would have asked more specific questions and focused only on the breach rather than letting her tell her story. (Frontline officer)

I have been practising using open-ended questions in the field every chance I get and found the questioning style to be really beneficial. It’s feeling more natural the more I do it. I did an interview about a week ago where I was Secondary. We were interviewing a suspect on a fraud case and the Primary, much like I would have been before doing the course, was asking so many specific questions. You could tell that the suspect knew about it but didn’t want to say anything. Anyway, I was just standing there . . . standing there . . . and finally I just spoke up and said to the suspect, ‘Tell us what you know about that’ and he did. I was like ‘Oh, this works!’ It was so good! The Primary kind of just looked at me in bewilderment because I got it out of him so easily – it was really cool. (Frontline officer)

### Learning outcomes could be improved

Some of the officers’ feedback consisted of recommendations for how learning could be improved. This feedback related to both the course activities and the level of organisational support during and after course completion. Regarding the course activities, the focus was on the mock interview exercises, in particular the realism of the response styles of the people who played the role of the witness or complainant. The perception was that variability in response style to different types of open-ended questions, and between open-ended and specific questions, was not as pronounced in the field than in the mock interviews. Some officers perceived that irrespective of the type of question, cooperative witnesses tend to provide elaborate detail, even to closed questions. A particularly frustrating response style in the mock interviews was when the role-players answered ‘Yes’ or ‘No’ to ‘Can you tell me about . . . ’ or ‘Do you remember’ questions.

The way that people speak normally in everyday life is very different to how you respond in the mock interviews. When I say, ‘can you tell me more about that part when this happened’, I say that because asking ‘can you’ sounds nicer than saying ‘tell me’ about this. So when I say ‘can you tell me . . . ’ they don’t just say yes or no, they would tell me and go through the whole entire story. Whereas in the mock interviews you would just say a couple of words and then stop I have to keep asking for more. This is not what happens in real life, they just tell you everything and they’ll go in-depth. So I find that these mock interviews aren’t very realistic and that made it hard to apply what I’ve been taught into practice (Frontline officer)

Not all the officers, however, agreed that the response style interfered with transfer of learning. Some perceived that the trained respondents’ style made them work hard to practise their skills, and this facilitated learning. Nonetheless, all feedback indicated that opportunities to practise were a priority for the police members and that they would have benefited from even more practice sessions, even at the cost of cutting back content. Having dynamic and vivid practice sessions was deemed important. Given the immense benefit they found in these tasks, it was a great source of frustration when they could not find the time within their heavy work schedules to get the activities done in a timely fashion.

I think there is always room for more practicals. Whilst time-consuming, the benefit of these cannot be underestimated. A face-to-face interview would be more realistic, as that’s how we do them in the field. (Detective)

Undertaking the course during work hours has limitations. It was difficult to have study days rostered then not being able to study because I was being interrupted or had to respond to an urgent event. The ability to undertake the course over an extended period of time is a good idea. My work–life balance has suffered due to insufficient rostered study days as I’ve had to complete the course in my own time. (Frontline officer)

Feedback provided by police related to the importance of course content being aligned with broader operational dialogue and procedures. Workplace supervisors who have not undergone the training were more likely to undervalue, and discourage the use of, questioning techniques that were taught in the course.

I came into this course with limited experience in relation to correct interview techniques. Traditionally I would sit in on an interview and observe the Senior Officer and integrate into my questioning what I thought worked well. After completing this course, I realise that even Senior Officers have ‘bad’ habits. I now have a new and improved way of questioning suspects, victims and witnesses. Before I was always focused on just getting the answer ‘I’ wanted from the suspect, and not allowing them to tell their version of events. I now know the importance of getting a free-flowing narrative from whoever I am interviewing and reducing the number of specific questions. (Frontline officer)

I’ve been getting really some good results with this new interview method. I’ve managed to get certain people talking that I wouldn’t have been able to before. Unfortunately, I have felt pressure from my Seniors to wrap it up, like ‘you’re going on a bit too much’, when I felt I was getting useful information. (Recruit)

Finally, the mismatch between the use of the term ‘interview’ in the training and field also caused some confusion. The course tended to use a broad inclusive term, including situations where questioning was conducted for the purpose of eliciting statements.

What the course called an ‘interview’ and what we call an interview are two completely different things. The course focused on interviews in the context of getting an account from a witness whereas our organisation refers to this as ‘taking statements’. (Frontline officers)

I question a lot of witnesses, but we don’t really call these interviews as such. We refer to them as statutory declarations. (Recruit)

## Discussion

The results of this study provide strong qualitative support for the benefit of the new investigative interviewer training programme and the usefulness of teaching the various subtypes of open-ended questions for generalist police work. All 155 officers felt that their ability to obtain narrative accounts had vastly improved since completing the course. Many officers also perceived that, due to their better use of open-ended questions, the quality and range of evidence obtained from witnesses and suspects had improved, and this would subsequently benefit prosecution.

Importantly, perception of the benefit of asking the open-ended questions was similar across all officers, despite large variability in their backgrounds, experience, personality (as noted anecdotally by the respondents who played the role of the witnesses) and whether they had received investigative interviewer training previously. While actual interview performance was not measured in this study, the strong support for the utility of open questions irrespective of individual factors is consistent with the conclusion of Smith et al. (2009) who examined the association between individual characteristics and interviewer performance. They found that training recency and quality accounted for most of the variability in interviewer performance whereas the effect of individual background factors was negligible. While there has been some debate in the literature about the impact of personality factors on interviewer performance (e.g. see Akca et al., 2021), it may well be that training quality mitigates those factors and needs to be factored into any interpretation of research results. Indeed, we know that competency in asking open-ended questions can minimise the detrimental effect of confirmation bias, which also differs markedly across individual officers (Powell et al., 2012).

The officers attributed their ability to adhere to open-ended questions to two main types of learning activities: the coding task where they needed to practise identifying different question types and the simulated practice opportunities (i.e. mock interviews). This is consistent with other qualitative training evaluation studies where police officers who have experienced these elements in training reported them to be beneficial (Lawrie et al., 2020; Wright & Powell, 2006), and those who had little practice opportunities and focus on questioning in their training have highlighted the need for more (Mount & Mazerolle, 2021). The experienced officers also attributed their better use of open-ended questions to the broader way in which the questions had been defined compared to previous courses. To date, the subtypes of open-ended questions defined in this study (i.e. open-ended invitations, open breadth questions and open depth questions) have been restricted to courses that focused on interviewing children. Most generalist police, including those involved in this study, were previously taught to elicit evidence mainly using ‘Wh’ (i.e. Who, What, When, Where, Why) questions, along with Tell, Describe, Explain questions (see Oxburgh et al., 2010, for review). According to the classification used in this study, none of those key words would define questions that elicited a narrative account. It is the variations of the phrase ‘ . . . what happened’ (i.e. ‘What happened then?’, ‘What happened when . . . ’ or ‘Tell me more about the part where . . . ’) that is most critical (Powell & Snow, 2007).

The officers’ strong support for the utility of the new classification of open-ended questions suggests that the ‘open-ended’ question classification system could account (at least in part) for the poor outcome of prior training evaluations that have examined the use of open-ended questions by generalist police. The need for careful reflection of the definition of open-ended questions has been recognised and discussed for some time by academic practitioners (Oxburgh et al., 2010; Westera et al., 2020), but so far there has been no direct comparison under controlled experimental conditions of the utility of the various classifications used in training programmes. Clearly, there is an urgent need to establish an empirical basis for recommendations, given the findings of this study and other research to date.

Another important issue for future research is consideration of the type of scenarios and response styles used in the simulated interviews. First, many of the officers appeared irritated that the respondent style in the simulated interviews was not consistent with how adults typically respond in the field. The concern is that the respondents were not giving sufficient information to the various questions and that this was distracting and inhibited their learning. A particular concern was the way the respondents answered yes or no to ‘Do you know/Can you tell me . . . ’. These questions are indirect speech acts because they indirectly ask respondents whether they know the answer to a question while indirectly seeking the content of the answer, if known (Clark, 1979). The officers argued that saying ‘yes’ or ‘no’ was unusual because most adults would understand the pragmatic intention behind the questions. Second, while most of the officers understood that open-ended questions could be potentially useful in any scenario type, some argued that because certain crime types (e.g. traffic accidents, theft) were *generally* less conducive to narrative detail than others (e.g. family and sexual violence), it was not appropriate to use these scenarios. As with the response style, the concern was that realism facilitated learning; because they did not tend to use open-ended questions in these scenarios, they felt disadvantaged by them.

Concern about the realism of interviews, however, is not limited to scenarios where research assistants play the role of adult interviewees. The reflections are like that reported in a study by Lawrie et al. (2020) where trainee police and social workers reflected on mock interviews where researchers played the role of the child. Their feedback tended to focus on the similarities between role-player and child, rather than how the role-play responses encouraged intended behaviours. Collectively, officers’ feedback raises questions about the elements or characteristics of investigative interview practice sessions that best guide learning. What we know so far is that simulated interviews with trained respondents is critical (Powell, 2008; Powell et al., 2008) and that for effective transfer of learning into the workplace, the simulation needs to *challenge* interviewers (i.e. presents them with interviewee response styles that make them most prone to deviate from best practice guidelines), and interviewers need to see that the open-ended questions are actually working (Powell et al., 2022; Powell et al., 2010; Sharman et al., 2012; Wright & Powell, 2006). Regardless of the scenario, the respondents had been trained to reward open-ended questions with detail, but not too much detail and specificity to invite further open-ended questions (Brubacher et al., 2014; Powell, 2008; Powell et al., 2008). The question for researchers is what level of realism is needed to best facilitate learning, and is there an inherent disadvantage from choosing certain scenarios as a standardised measure of police interviewer performance? Once the empirical basis for making decisions has been firmly established, the goal and structure of the sessions and their role in facilitating learning need to be explained to trainee interviewers so they are in the right mindset to get the best out of these sessions.

Recommendations can also be made for how police organisations support the police trainees to get the most out of the training programme. As with previous training evaluations conducted in other jurisdictions (e.g. Benson & Powell 2015; Wright & Powell, 2006; Wright et al., 2006), ensuring that supervisors’ expectations and skills are aligned with the protocols taught in training is important, along with ensuring sufficient time in police workloads to complete the course. Although time was allocated, this course was time consuming due to the nature of the activities (e.g. scheduling and conducting mock interviews) and structure, and therefore the qualitative feedback may not be applicable to other courses with different content and structure. Urgent work sometimes took priority, and learning that was rushed was deemed to compromise skill development. This view is consistent with the prior literature on interview training, which has shown that practice needs to be spaced (interspersed with rest intervals) and maintained over a long period of time (see Powell, 2008, for review).

Further, the results suggest that it would be beneficial to develop evidence-based guidance around when open-ended questions may and may not be useful for frontline generalist police officers. At present, decisions around when to use open-ended questions tend to be made through trial and error, and there is marked variability in perceptions among individual officers regarding whether there is a place for open-ended questions in certain case types (e.g. traffic accidents, burglary, fraud). Improved consistency and clarity around the investigative requirements and process could evolve through research like that conducted by Burrows and colleagues (for example, Burrows & Powell, 2014, 2015). In collaboration with key stakeholders, Burrows conducted a series of focus groups using case scenarios to develop (through thematic analysis) guidelines around when to ask follow-up questions in interviews with child sexual abuse complainants. The instruction they developed led to a shift in decision making that was more consistently aligned with organisational goals (Burrows et al., 2013).

In conclusion, this study has added to a growing call in the literature for better consideration of the way open-ended questions are defined, taught and promoted in generalist police training programmes (Oxburgh et al., 2010; Westera et al., 2020). Although police officers once viewed interviewing as a ‘wisdom that comes naturally to professionals through their job experience’ (Cioccarelli, 1989, p. 40), this attitude has shifted through better understanding of how expertise in interviewing is learnt and sustained. The officers in the current study perceived interviewing as a highly specialised skill that requires training and monitoring of performance, through practice and critical feedback in the use of open-ended questions.

## Appendix: Overview of the adult interviewing training programme.

**Table:**

Module title	Learning outcomes	Description of activities
Best-practice guidelines^a^	Understand the impact of various question styles. Identify the elements and benefits of an effective interview training programme.	Interview colleague to experience and reflect on various questioning styles from an interviewee’s perspective. Commentaries and film (the ‘Massage Film’) of an implied sexual assault, highlighting the impact of various questions. Reading on rationale and focus of the course with corresponding quiz to test understanding.
Defining the questions^a^	Distinguish between open-ended versus specific questions and non-leading versus leading questions. Recognise the various subtypes of open-ended questions. Identify which minimal encouragers are effective in facilitating the use of open-ended questions. Label (to a high degree of reliability) various questions in an interview transcript.	Learning of coding protocol (question types and subtypes). Coding mock interview transcripts of various offence types (quiz format with immediate feedback).
Understanding memory and behaviour	Describe how memory operates. Demonstrate interview strategies to minimise errors or misunderstandings. Identify how misconceptions about memory and behaviour can influence the interview process.	Narrated PowerPoint lecture on memory. Reading and videos on memory and susceptibility to error. ‘Truth or Lie’ filmed videos with associated activities highlighting the difficulty with deception detection (misconception).
Practicing the right questions to elicit a narrative^a^	Automatically retrieve the various types of open-ended questions that can be used in an interview. Insert ground rules into appropriate places throughout the interview. Incorporate minimal encouragers into your interviewing technique.	Rote learning activities to practice and memorise non-leading open-ended stems. Informal interview practice with a colleague to incorporate breadth and depth prompts with minimal encouragers and ground rules. Commentaries and transcripts of people being interviewed about the ‘Massage Film’, highlighting the effect of various questions. Reading about the nature of the limitations of open-ended question usage. Podcast from Police (Summary for Police).
Raising the topic of concern^a^	Know which techniques are most useful in eliciting disclosures of alleged offences. Generate questions that may be useful (in certain case scenarios) for raising the topic of concern and eliciting disclosures. Know how to plan effectively for an interview.	A guide on how to introduce the topic of an interview for various contexts. Activities on developing questions to introduce the topic of concern. Podcast from Police (Planning and Control). 4 × best-practice exemplar films and associated activities on how to raise the topic of concern (various offence types). Mock interview practice with trained role-player (includes immediate verbal feedback).
Assessing your progress^a^	Identify how your performance has improved in this course to date.	Electronically recorded mock interview with trained role-player playing the interviewee of an alleged offence. Exercise to transcribe, code and reflect on own interview. Student receives written feedback on questioning and coding accuracy.
Interview protocols and repeated events^a^	Learn the challenges associated with interviewing witnesses about repeated experiences. Outline the key components of the SIM-Adult-1 tool. Competently administer the SIM-Adult-1 in a mock interview setting, Apply the SIM-Adult-1 when interviewing about repeated experiences.	Reading of the interview protocol and the rationale behind the development of the protocol. Readings on generic vs. episodic questioning accompanied by transcripts and commentaries identifying the effects of various questioning styles. Mock interview with trained role-player using the interview protocol (includes immediate verbal feedback).
Overcoming language barriers	Understand common pitfalls of complex communication. Use techniques to improve simple communication appropriate for all interviewees.	Film defining complex communication and outlines common issues that complicate conversational exchanges and increase misunderstanding. Exercise to simplify complex questions.
Policy recording and documentation	Identify Police processes in respect of obtaining an account from a witness. Develop strategies for taking notes during an interview. Take notes from an account provided and covert that into a first-person chronological statement.	Exercises to practice converting police notes from a verbal narrative into a police statement (statutory declaration). Mock interview with trained role-player incorporating appropriate specific questions (includes immediate verbal feedback).
Suspect interviews – History and the law	Identify, understand and apply legislation governing interviews with suspects. Understand the rationale for legislative protections for suspects during interviews with investigating officials. Explain the benefits and methods for developing rapport with a suspect. Identify appropriate tactics to assist in overcoming deception. Outline the legislative and Police requirements when conducting an interview with a suspect. Plan for a suspect interview.	Narrated PowerPoint lectures and quizzes on the history and law of police legislation. Narrated PowerPoints and associated quizzes to check understanding on suspect interviewing procedures specific to police. Reading of the Suspect Interview protocol
Putting it all together	Demonstrate understanding and knowledge of the information presented in the previous 10 modules. Evaluate the effectiveness of an interview by discussing questioning techniques used. Demonstrate adherence to best-practice guidelines of interviewing adult witnesses.	Full mock interview trained role-player (written feedback provided). Quizzes on coding and previous modules. Written evaluation of a victim interview transcript.

*Note:* SIM = Standard Interview Method.

^a^Modules containing core learning elements known to promote sustained learning of open-ended questions. Adapted from Zekiroski, H., Powell, M., Brubacher, S., & Curtis, P. (2024). Evaluation of a police training program designed to enhance open-ended questions with adult witnesses. *International Journal of Police Science and Management*, 1–13. March 04, 2024. CC-BY 4.0. Sage Publisher. https://doi.org/10.1177/14613557241233857
